# Introducing blueberry powder as one of the first complementary foods changes the gut microbiota composition and diversity in U.S. human milk-fed infants: a double-blind, randomized controlled trial

**DOI:** 10.3389/fnut.2025.1623521

**Published:** 2025-09-04

**Authors:** Gabrielle N. E. Glime, Kinzie L. Matzeller, Daniel N. Frank, Cassandra Kotter, Jennifer M. Kofonow, Charles E. Robertson, Carina Venter, Wayne W. Campbell, Nancy F. Krebs, Minghua Tang

**Affiliations:** ^1^Department of Pediatrics, Section of Nutrition, University of Colorado Anschutz Medical Campus, Aurora, CO, United States; ^2^Department of Food Science and Human Nutrition, Colorado State University, Fort Collins, CO, United States; ^3^Department of Medicine, Division of Infectious Diseases, University of Colorado Anschutz Medical Campus, Aurora, CO, United States; ^4^Section of Allergy and Immunology, Department of Pediatrics, University of Colorado Anschutz Medical Campus, Aurora, CO, United States; ^5^Department of Nutrition Science, Purdue University, West Lafayette, IN, United States

**Keywords:** blueberry, gut microbiome, complementary food, infant feeding, infant nutrition, growth

## Abstract

**Introduction:**

Complementary feeding is a critical period marked by rapid changes in the infant’s diet, nutrient needs, and gut microbiota. However, the effects of specific foods introduced during complementary feeding, such as blueberries, on the developing infant gut microbiota remain unclear. Our primary aim was to evaluate the effect of daily consumption of freeze-dried blueberry powder during complementary feeding on gut microbiota development in U. S. human-milk-fed infants.

**Methods:**

In a double-blind, randomized, placebo-controlled feeding trial, infants from the Denver metro area (Colorado, United States) were randomly assigned to consume up to 10 g of freeze-dried blueberry powder or an isocaloric placebo powder, combined with liquid or semi-liquid and served as a puree, daily from 5 to 12 months of age. Stool samples were collected bimonthly to assess gut microbial diversity and composition. Novel taxa were identified through parsimony insertion into the SILVA reference phylogenetic tree. Infant length, weight and dietary intakes were also assessed.

**Results:**

Seventy-six caregiver-infant pairs consented and enrolled in the study and 61 completed the study (blueberry group *n* = 30, placebo group *n* = 31). There were no differences between groups in energy or macronutrient intakes from complementary foods. Growth *z*-scores were comparable between groups. Gut microbiota alpha diversity increased over time in both groups (effect of time *p* < 0.001). Several taxa, including *Veillonaceae, Flavonifractor, Subdoligranulum,* and *Butryicicoccus* (all more abundant in the blueberry group), and *Actinomyces, Escherichia, Streptococcus,* and *Romboutsia* (more abundant in the placebo group) had group-by-time interactions that trended toward significance.

**Conclusion:**

Introducing blueberries as one of the first complementary foods, in the form of freeze-dried powder served as a puree, exerts potential benefits in gut microbiota development and maturation in this cohort of human-milk-fed infants.

**Systematic review registration:**

https://clinicaltrials.gov/study/NCT05006989.

## Introduction

The transition from the liquid diet (e.g., human milk, infant formula) to the introduction of solid foods, referred to as complementary feeding, normally starts around 6 months of age, when an infant is developmentally ready, as recommended by the World Health Organization and the American Academy of Pediatrics (1, 2). Complementary feeding is considered a critical developmental phase for infants, marked by rapid changes in dietary intake, nutrient needs, and the gut microbiota. This dynamic and malleable phase is critical for shaping the gut microbiota composition, which remains relatively stable from late childhood through adulthood (3), but is immature and more susceptible to modulation during infancy (4). Early manipulation of the gut microbiota has the potential to influence the developing immune system and long-term health outcomes and disease risks, including allergy, type 1 diabetes, obesity, and asthma (5, 6). Thus, complementary feeding presents a unique opportunity to promote the development of a healthier gut microbiota, which may confer long-term health benefits.

While diet influences the adult gut microbiota, a critical gap exists in understanding how specific foods introduced during complementary feeding influence infant gut microbiota development. Emerging evidence suggests that complementary foods can alter gut microbiota composition (7–9). For example, meat-and dairy-based complementary foods led to different gut microbial diversity and composition in formula-fed infants (10). The current consensus holds that the introduction of complementary foods increases gut microbial diversity and alters the abundances of certain taxa, such as *Bifidobacterium* (6, 8). Additionally, powdered fruit and vegetables have been shown to affect the relative abundance of bacterial families *in vitro*, including *Prevotellaceae*, *Enterococcaceae, and Lactobacillaceae* (11). Furthermore, blueberries are a good source of prebiotic compounds such as anthocyanins and fiber (12). Animal studies indicate that blueberry consumption can promote the growth of commensals (13) and restore impaired gut integrity (14). In adults, daily blueberry consumption has been linked to increased levels of beneficial *Bifidobacterium* spp. (15). A recent systematic review summarized, based primarily on animal studies, that blueberry improves gut microbial diversity and commensal abundances (16). Despite these promising findings, a paucity of research exists investigating the effect of blueberries during complementary feeding on infant gut microbiota.

We conducted a double-blind, randomized controlled feeding trial to evaluate the effect of introducing blueberry powder (served as a puree) as one of the first complementary foods (solid food) on gut microbiota development in human-milk-fed infants. The central hypothesis was that the inclusion of blueberry powders in an infant’s complementary diet would favorably change the gut microbiota. Specifically, compared to the placebo group, the blueberry group would have higher gut microbial diversity and greater abundances of potentially beneficial microbes. Moreover, adding blueberry powder into the complementary diet was not expected to change the infants’ overall dietary intakes or physical growth.

## Methods

### Participants

This was a double-blind, randomized, placebo-controlled feeding trial examining the effects of daily freeze-dried blueberry powder consumption on gut microbiota development in human-milk-fed infants. Households in the metro Denver area (Colorado, United States) with 3-4-month-old infants were contacted via direct mailing with the study flyer by the Colorado Department of Public Health & Environment (CDPHE). Flyers were also posted at local libraries, gyms, and shared within local breastfeeding mother groups. Infants were eligible to participate if they met the inclusion criteria: (a) Full-term birth (≥ 37 weeks gestation); (b) generally healthy without conditions that would affect growth; (c) minimal previous complementary food exposure (less than one ounce weekly); (d) human-milk-fed at enrollment (less than 2 weeks of cumulative formula exposure); (e) no prior exposure to antibiotics during delivery or after birth or pre−/pro-biotics after birth; and (f) able and willing to consume study foods (blueberry or placebo powder). The cut-off for formula exposure prior to enrollment was calculated as the number of days the infant consumed mostly formula (e.g., <14 days of the infant’s diet coming from mostly formula); see Dietary Intake for more information on the percentage of participants with formula exposure prior to enrollment. Infants who did not meet the prior inclusion criteria were excluded from the study, however infants who were introduced to formula during the intervention were not removed from the study. Complementary foods were defined as any food which is not human milk at enrollment.

After enrollment, participants were randomized using block randomization to either a blueberry group or a placebo group using Excel-generated random numbers. Blocks were defined by the infant’s sex and mode of delivery and were filled as participants enrolled. To maintain the double-blinded study design, groups were defined as group “A” and “B” for the blueberry and placebo groups, respectively. Investigators were unblinded after all the study related sample and data analysis were completed. This study was approved by the Colorado Multiple Institutional Review Board (COMIRB # 20–1,659) and registered at clinicaltrials.gov (NCT05006989) on July 19, 2021. Participants received monetary compensation for each study visit.

### Study design

The first (baseline) study visit occurred between 5 and 6 months of age. Participants and their caregiver(s) visited the Clinical and Translational Research Center (CTRC) at Children’s Hospital Colorado (CHCO) to complete the initial procedures, including obtaining informed consent from the participant’s caregiver followed by a baseline questionnaire which covered feeding and family health history, family demographics, parental weight and height, gestational weight gain, medication use during pregnancy, parity, maternal smoking and alcohol use, history of allergies using the International Study of Asthma and Allergies in Childhood questionnaire, stooling patterns, and other variables which could affect the study’s primary outcomes. Anthropometric measurements were also obtained at this visit in triplicate to obtain an average measurement. Infant blood and stool samples were also collected.

Three home visits were conducted at 7, 9, and 11 months of age (±7 days); each visit consisted of a health and allergy questionnaire (data not shown), anthropometric measurements, infant stool sample collections, a three-day diet record and study food intake log, and delivery of the blueberry or placebo powder. The food intake log asked caregivers to record daily blueberry/placebo powder consumption through a multiple-choice question. Additional home visits were made upon the caregiver’s request to deliver additional blueberry or placebo powder if used, lost, or contaminated. Upon completion of the study at 12 months of age, participants returned to the research facility at CHCO CTRC for the end-of-study visit. The baseline visit procedures were repeated with the addition of collecting any remaining logs from the study participants.

At each visit, participants were provided with pre-packaged 10 g packets of their assigned group powder. The blueberry group received freeze-dried highbush blueberry powder, which was equivalent to approximately two ounces of fresh blueberries or five infant servings of fruit (17, 18). The placebo group received identical packets of an isocaloric placebo powder. A blueberry powder was used in place of fresh blueberries, as whole blueberries present a choking hazard for young children. A powdered form of blueberries also allowed for the study to be double blind with the use of a placebo powder. Nutrient breakdown of each powder is provided in Supplementary Table 1. The U. S. Highbush Blueberry Council provided both the freeze-dried blueberry powder (same harvest) and the placebo powder. Blueberry/placebo packets were to be stored in the participant’s home freezer at −20°C before consumption. Caregivers received recommendations from the study team on how to introduce the powder, including mixing with human milk, formula (if introduced), other fruit and vegetable purees, yogurt, and infant cereal. The powder forms puree when mixed with liquids according to the study instructions and is thus fed as a puree rather than a powder. Parents and caregivers were asked to offer up to one packet per day from baseline until 12 months; each packet could be offered in one sitting or throughout the day at multiple mealtimes. Caregivers were instructed to record the infant’s packet consumption on a provided intake log; this log included spaces to note the preparation method, time(s) of consumption, and any additional infant and maternal consumption of blueberries and blackberries. The daily intake log also had a multiple-choice question asking if the participant consumed: all, more than half, half, less than half, or not much of the packet. The intake logs were used to calculate compliance as the average percentage of one packet per day.

### Dietary intake and growth assessment

Caregivers were instructed to complete 3-day diet records before each study visit. Records were collected on consecutive days, including two weekdays and one weekend day; when possible, it was requested that parents avoid days when the infant was in childcare due to the risks of inaccurate recording by childcare staff. While participants were exclusively human-milk-fed at enrollment (<14 days of the infant’s calories coming primarily from formula), the introduction of formula did not exclude them from participating in the remainder of the study. Data regarding formula usage were obtained at study visits and through diet records. Expressed human milk was recorded in ounces (bottles) and as duration of a nursed feed (minutes, which breast). Formula and solid foods were recorded by quantity consumed. Dietary intake data of solid foods were analyzed using Nutrition Data System for Research software version 2024, developed by the Nutrition Coordinating Center (NCC), University of Minnesota, Minneapolis, MN. Average daily intake values were reported at three timepoints: baseline, 9 months (midpoint), and 12 months (endpoint).

Because the blueberry and placebo powders had unique nutrient profiles not included in the NDSR database, their nutritional contributions were calculated separately. The study food logs completed by participants were used to calculate their nutrient intakes using provided Nutrient Facts panels from the United States Highbush Blueberry Council. Calculated values were then manually added to the NDSR output to improve the accuracy of reported nutrient intakes from study powder. As direct quantification of human milk intake during nursed breastfeeding sessions was not feasible with the diet records collected, human milk and formula intake were excluded from the dietary analysis. Therefore, this analysis focuses on the intake of quantified complementary foods and the blueberry/placebo powders. Mode of feeding information is presented separately in the results section.

Anthropometric measurements of weight, length, and head circumference were obtained at each visit. For the baseline and 12-month visits, these measurements were completed by pediatric research nurses at the CHCO CTRC. Measurements completed during home visits occurring at 7, 9, and 11 months were conducted by the same trained study coordinator (GG). Z-scores for weight, length, and head circumference were calculated using WHO growth chart standards for infants 0–24 months (19).

### Gut microbiota profiling

Caregivers were instructed to collect fecal material from a soiled diaper from their infant at each visit. Disposable diapers fitted with biodegradable liners (provided) were used to collect stool samples, which were collected from participants’ homes by caregivers and temporarily stored in home freezers at −20°C. The samples were then picked up and transported back to the laboratory on ice packs by study coordinators. Samples were processed in a PCR hood using single-use gloves and sterile sampling spoons, then stored at −80°C until analysis.

Bacterial profiles were determined by broad-range amplification and sequence analysis of 16S rRNA genes following our previously described methods (10, 20, 21). In brief, DNA was extracted from 25 to 50 mg of stool using the QIAamp PowerFecal DNA kit (Qiagen Inc., Carlsbad, CA), which employs chemical and mechanical disruption of biomass. Samples were bead-beaten using a MagNA Lyser (Roche Inc., Basel, Switzerland) at 10,000 rpm for 60 s. PCR amplicons were generated using barcoded (22) primers that target approximately 450 basepairs of the V3V4 variable region of the 16S rRNA gene (338F: 5’ACTCCTACGGGAGGCAGCAG and 806R: 5’ GGACTACHVGGGTWTCTAAT) (23, 24). PCR products were normalized using a SequalPrep™ kit (Invitrogen, Carlsbad, CA) and then pooled. The amplicon pool was partially lyophilized to reduce its volume then purified and concentrated using a DNA Clean and Concentrator Kit (Zymo, Irvine, CA). Pooled amplicons were quantified using a Qubit Fluorometer 2.0 (Invitrogen, Carlsbad, CA). Illumina paired-end sequencing was performed following the manufacturer’s protocol on the MiSeq platform using a 600-cycle version 3 reagent kit and version v2.4 of the MiSeq Control Software.

Paired-end reads were aligned to human reference genome hg19 with bowtie2 and matching sequences discarded (25, 26). As previously described (10, 20, 21), demultiplexed paired reads were assembled using phrap (27, 28) and pairs that did not assemble were discarded. Assembled sequences were trimmed over a moving window of 5 nucleotides until average quality met or exceeded 20. Trimmed sequences with more than 1 ambiguity or shorter than 350 nt were discarded. Potential chimeras identified with Uchime (usearch6.0.203_i86linux32) (29) using the Schloss (30) Silva reference sequences were removed from subsequent analyses. Assembled sequences were aligned and classified with SINA (1.3.0-r23838) (31) using the 510,508 sequences in Silva 138.1NR99 (32) as reference configured to yield the Silva taxonomy. Taxonomic assignment by SINA used the lowest common ancestor approach with default parameters. Operational taxonomic units (OTUs) were produced by binning sequences with identical taxonomic assignments; groups not classified to the genus level were appended with “_other” (e.g., sequences classified only to the family “Veillonellaceae” rather than the genus “Veillonella” were designated “Veillonellaceae_other”). All 316 sequence libraries contained >10,000 16S rRNA gene sequences after filtering and classification steps (median of 61,001.5 sequences/sample; IQR: 37,272.5 – 95,854) and all libraries had Good’s coverage values >99%. The software package Explicet (v3.3.22) (33) was used to calculate alpha diversity indices, through 1,000 replicate re-samplings at a rarefaction point of 10,000 reads. Sorted paired end sequence data along with associated metadata were deposited in the NCBI Sequence Read Archive under accession number PRJNA1245476.

### Statistical analysis

The software package R (v 2024.12.1 + 563) was used to analyze data. Participant demographics and growth *z*-scores between the blueberry and placebo groups were compared using chi-square tests for categorical variables and independent *t*-tests for quantitative data. When comparing growth between groups longitudinally, a linear mixed-effects model was used, which included gender, formula consumption, and timepoint.

For microbiota analysis, alpha-diversity indices (richness [Sobs], diversity [Shannon H], and evenness [Shannon *H*/Hmax]) were assessed by linear mixed effects models (lmer function of lme4 package (34)). Community-wide differences in overall composition (i.e., beta-diversity) were assessed through GLMM-MiRKAT (35) or PermanovaG2 (36) R functions for longitudinal or cross-sectional analyses, respectively. Kernel matrices were derived from Aitchison, Bray-Curtis, and Jaccard dissimilarity matrices, along with generalized UniFrac (36) dissimilarity matrices generated with alpha values of 0, 0.5, and 1. *p*-values were inferred through 10 (5) label permutations. Principal coordinates analysis (PCoA) was carried out using Aitchison dissimilarities and the vegan wcmdscale function. Individual taxa with statistically significant Group*Age interactions were identified using the lmertest (37) R packages for linear mixed-effects modeling. The distribution of taxa in each sequence library was estimated through 250 Dirichlet Monte Carlo re-samplings of sequence count data (38, 39). To account for the compositional nature of microbiome data (40), sequence count data were subjected to a centered log-ratio (CLR) transformation with all features used as the denominator. For all microbiota analyses, actual or FDR-corrected (41) *p*-values are reported, as indicated in the text and figures. Due to the exploratory nature of the microbiome study, significance was assessed at *p* = 0.1.

Differences in dietary intake between the blueberry and placebo groups were analyzed using independent *t*-tests in a cross-sectional analysis. Comparisons were conducted separately at each time point (baseline, 9 months, and 12 months) to assess whether dietary intake varied between groups throughout the study. Given the examination of multiple dietary variables, Bonferroni’s correction was applied. With eight dietary variables tested, the adjusted significance threshold was set at *p* < 0.006 (0.05/8). To assess growth between groups, we compared anthropometric z-scores cross-sectionally using independent sample *t*-tests, and longitudinally with a linear mixed-effects model to examine the relationship between growth and time. Time was treated as a continuous variable in the model with subjects as random intercepts. A likelihood ratio test was used to compare models with and without the interaction term. After accounting for multiple testing using Bonferroni’s correction, the adjusted significance threshold was set at *p* < 0.01 for growth differences. Formula consumption at baseline and 12 months between groups was assessed using independent *t*-tests.

## Results

### Participants

**Figure 1:**
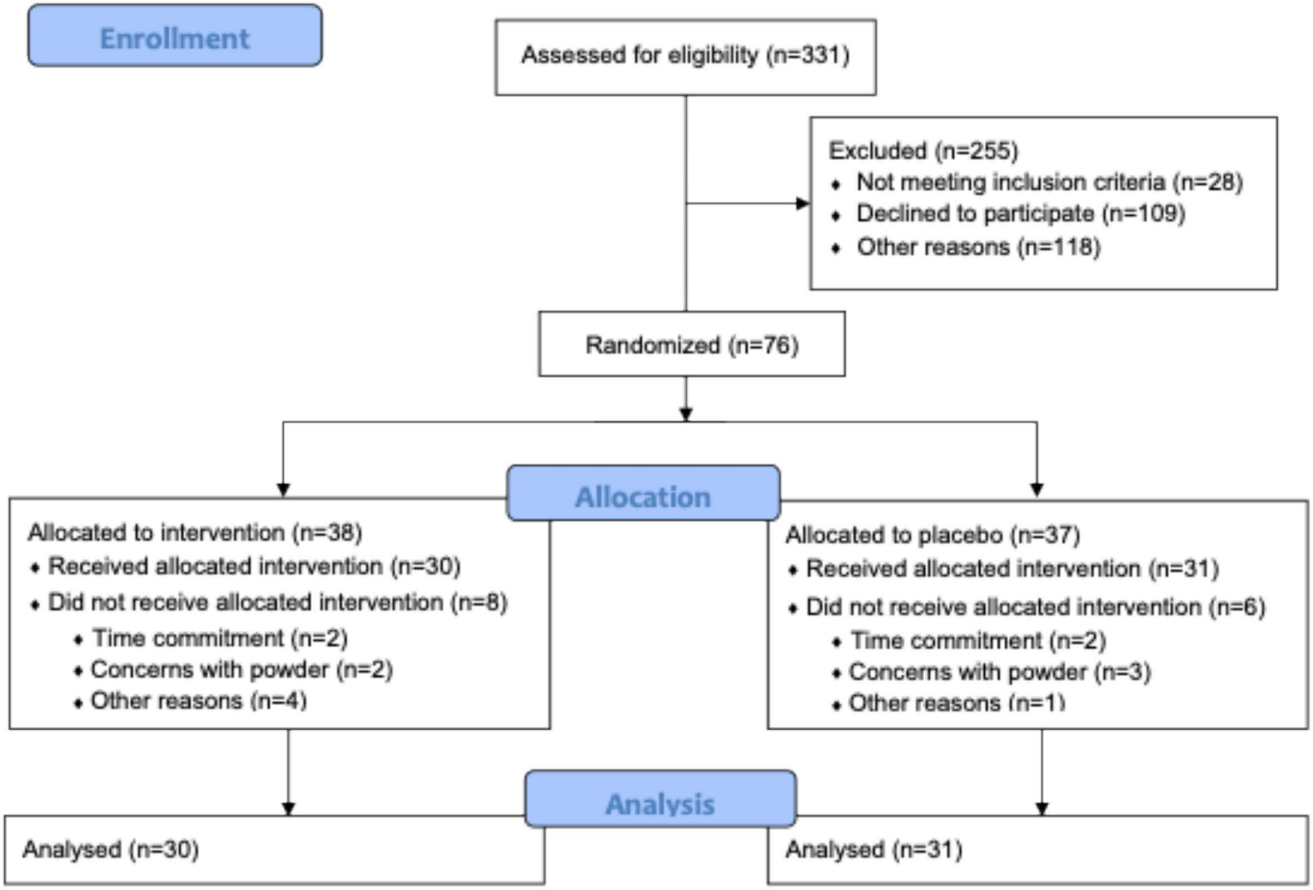
Consort diagram.

A total of 331 contacts were received after flyers were posted and mailed, and 76 infants were enrolled in the study. Sixty one participants completed the study (blueberry group *n* = 30, placebo group *n* = 31) (Figure 1). Demographic results for study participants are presented in Table 1. Groups were referred to as A (blueberry group) and B (placebo group) until all data analysis was completed. Percent compliance differed between groups, with the placebo group having a higher percent compliance at 11 months (60.6% vs. 37.4%, *p* = 0.003) and 12 months (61.1% vs. 36.8%, *p* = 0.004).

**Table 1:** Demographic data for the 61 participants who completed the 7-month study.

Characteristics	All participants (*n* = 61)	Blueberry group (*n* = 30)	Placebo group (*n* = 31)	*p*-value
Sex (M), *n* (%)	36 (59)	21 (70)	15 (48)	0.08
Race (White), *n* (%)	50 (82)	24 (80)	26 (84)	0.15
Ethnicity (non-Hispanic), *n* (%)	49 (80)	28 (93)	21 (68)	**0.009**
Gross family income, *n* (%)				
<$100,000	28 (47)	9 (30)	19 (63)	**0.03**
$100,000–199,000	20 (33)	13 (43)	7 (23)	
>$200,000	12 (20)	8 (27)	4 (13)	
Maternal education, *n* (%)				
High school	4 (7)	1 (3)	3 (10)	0.22
Some college	6 (10)	4 (14)	2 (7)	
Associate’s degree (2 yr)	5 (8)	1 (3)	4 (13)	
Bachelor’s degree (4 yr)	19 (32)	8 (28)	11 (36)	
Master’s degree	20 (33)	10 (35)	10 (32)	
Doctorate/professional degree	6 (10)	5 (17)	1 (3)	
Parity, n ± SD	1.83 ± 0.8	1.67 ± 0.8	2 ± 0.9	0.12
Maternal BMI, kg/m^2^ ± SD	26.7 ± 6.7	25.7 ± 7.1	27.7 ± 6.2	0.25
Mode of delivery (vaginal), *n* (%)	47 (77)	23 (77)	24 (77)	0.94
Percent powder consumed at 12-months, mean ± SD	49.4 ± 31.1	36.8 ± 29.9	61.1 ± 28.8	**0.004**

For sex, race, ethnicity, gross family income, maternal education, and mode of delivery, data are presented as mean (percent). Parity, maternal body mass index (BMI), and percent adherence are presented as mean ± standard deviation. Significant differences are shown in bold (*p* < 0.05). SD: standard deviation.

### Dietary intake

Dietary intake results reflect solid food intake (and exclude human milk and formula intakes) at 5, 9, and 12 months. The sample size for 5 months was low (*n* = 5, blueberry group *n* = 3 and placebo group *n* = 2), as most participants had not yet started solid foods; 70% of the infants had never had any formula from birth to enrollment, and of those that had, 70% had exposure for less than 1 week and prior to enrollment. At 9 and 12 months, dietary intake data (excluding human milk and formula) were analyzed for *n* = 39 participants (blueberry group *n* = 20, placebo group *n* = 19) for whom complete 3-day diet records were returned. Diet records without three consecutive days of intake data were excluded from this analysis.

Based on 3-day diet records and compliance data for study food powder, no significant differences were observed at any time point between groups for energy, macronutrients, or fiber intakes. When considering the mode of feeding, the percentage of participants receiving human milk did not differ between groups during the study. Although formula intake was not included in Table 2, a separate formula intake analysis was conducted at 12 months between groups. The blueberry group had a non-significantly higher percentage of infants consuming formula (*p* = 0.14) at 12 months, and no differences in formula volume between groups were detected (*p* = 0.44). These analyses can be found in Supplementary Table 2.

**Table 2:** Average nutrient intake between groups at 5, 9, and 12 months.

Nutrient	Visit (month)	Blueberry (*n* = 30)		Placebo (*n* = 31)		*p*-value
		Mean	95% CI	Mean	95% CI	
Energy (kcal/day)	5	128	[0, 269]	41	[15, 68]	0.18
	9	297	[239, 356]	235	[185, 284]	0.10
	12	543	[461, 626]	527	[430, 624]	0.79
Energy (kcal/kg)	5	20	[0, 46]	6	[2, 10]	0.21
	9	36	[29, 43]	29	[23, 35]	0.11
	12	59	[51, 68]	60	[49, 72]	0.89
Carbohydrates (g)	5	21	[0, 44]	7	[3, 11]	0.18
	9	44	[35, 53]	38	[30, 45]	0.25
	12	72	[62, 83]	69	[56, 81]	0.63
Protein (g)	5	4	[0, 8]	1	[0, 2]	0.14
	9	10	[8, 13]	7	[5, 9]	0.05
	12	22	[18, 26]	22	[18, 26]	0.94
Fat (g)	5	4	[0, 9]	1	[0, 2]	0.24
	9	10	[7, 12]	7	[5, 9]	0.09
	12	19	[15, 23]	19	[15, 24]	0.97
Total fiber (g)	5	2	[0, 5]	1	[0, 2]	0.30
	9	5	[4, 6]	4	[3, 5]	0.17
	12	7	[6, 8]	6	[5, 8]	0.41
Soluble fiber (g)	5	1	[0, 1]	0	[0, 1]	0.40
	9	1	[1, 2]	1	[1, 1]	0.60
	12	2	[1, 2]	2	[1, 2]	0.82
Insoluble fiber (g)	5	2	[0, 4]	1	[0, 1]	0.26
	9	3	[2, 4]	3	[2, 4]	0.99
	12	4	[3, 5]	4	[3, 5]	0.92

Table reflects intake of selected nutrients at baseline (5 months), study midpoint (9 months), and endpoint (12 months). After applying Bonferroni’s correction, significance was determined to be *p* < 0.006. Sample size differed between groups and time points. 5 months: *n* = 5 (blueberry *n* = 3, placebo *n* = 2); 9 and 12 months: *n* = 39 (blueberry *n* = 20, placebo *n* = 19).

### Growth

**Figure 2:**
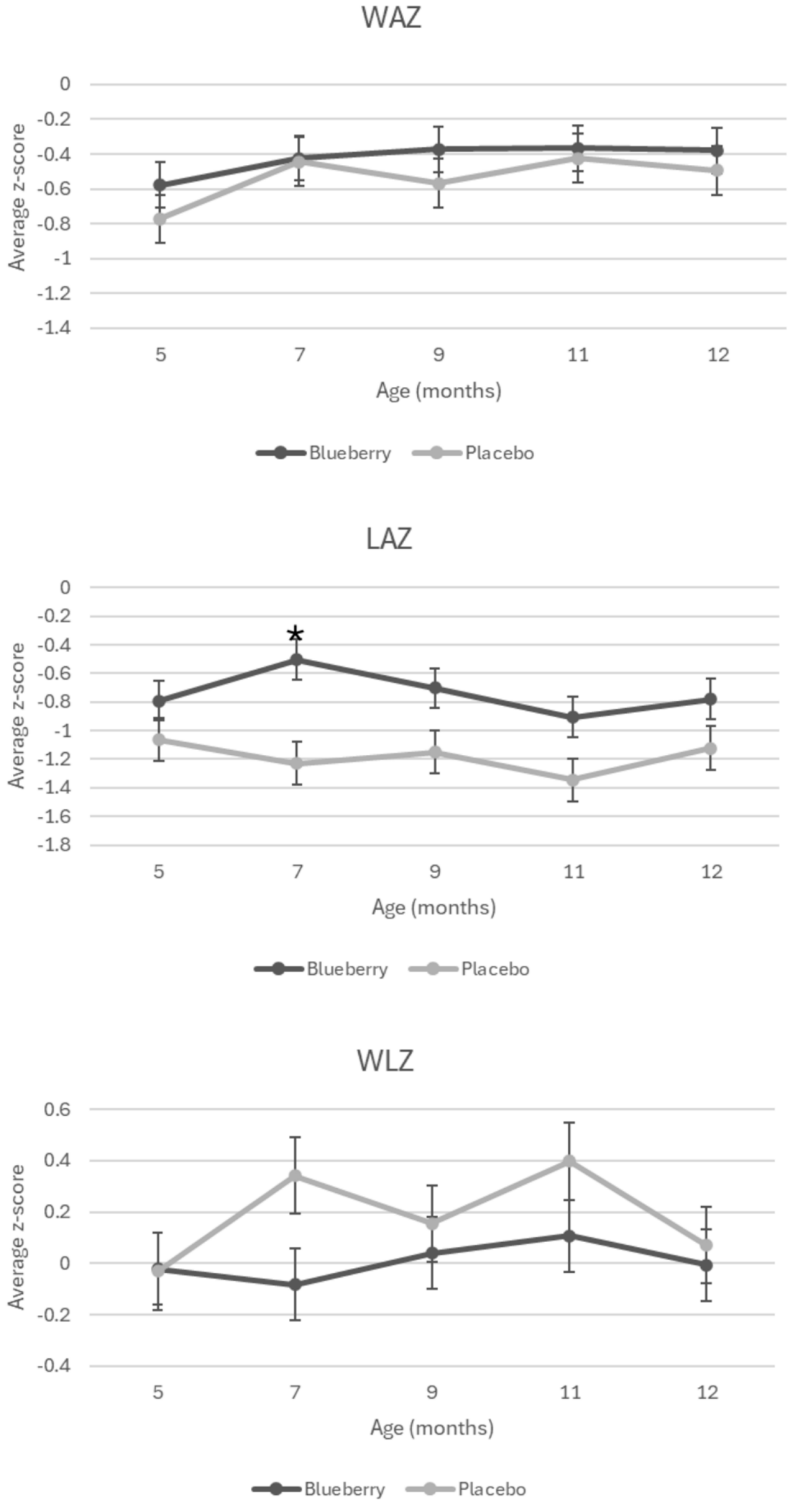
Growth *Z* scores over time and between the blueberry and placebo groups. Figure shows average *z*-score measurements at each time point during the study by group. Significant difference denoted by *. WAZ: weight-for-age z-score. LAZ: length-for-age *z*-score. WLZ: weight-for-length *z*-score.

When each visit was compared cross-sectionally between groups, no significant differences were seen between groups for weight-for-age *z*-scores (WAZ), length-for-age (LAZ), weight-for-length *z*-scores (WLZ), or head circumference-for-age z-scores (HCAZ). Average *z*-scores for WAZ, WLZ, and LAZ for each group are presented in Figure 2. While one time point (7 months) showed differences between groups in length-for-age *z*-scores (LAZ) with the blueberry group exhibiting greater LAZ (*p* = 0.004), this difference did not persist over the remaining visits. When compared longitudinally, using a linear mixed-effects model to evaluate changes in growth over time between groups with sex, formula, and timepoint included, no differences were observed between groups for any growth *Z* scores. However, throughout the study, participants exhibited an overall lower than population median WAZ (−0.49) and LAZ (−0.96).

### Gut microbial diversity

**Figure 3:**
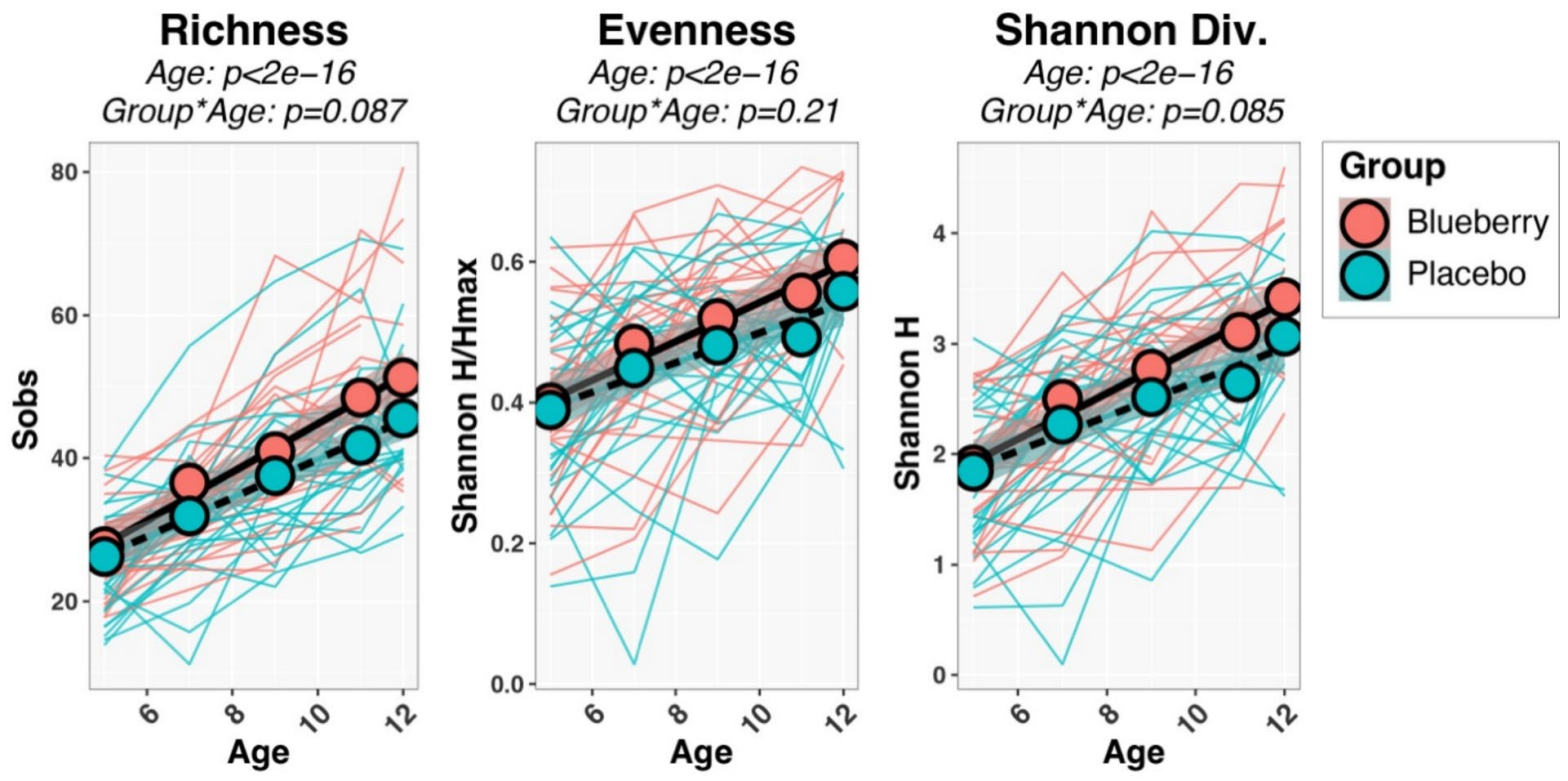
Longitudinal changes in alpha-diversity indices. Spaghetti plots show the development of each infant’s alpha-diversity scores (thin lines), while group means are shown by circles and thicker lines. *p*-values were inferred through repeated-measures regression of Age, Group, and Age*Group, with SubjectID included as a random effect.

**Figure 4:**
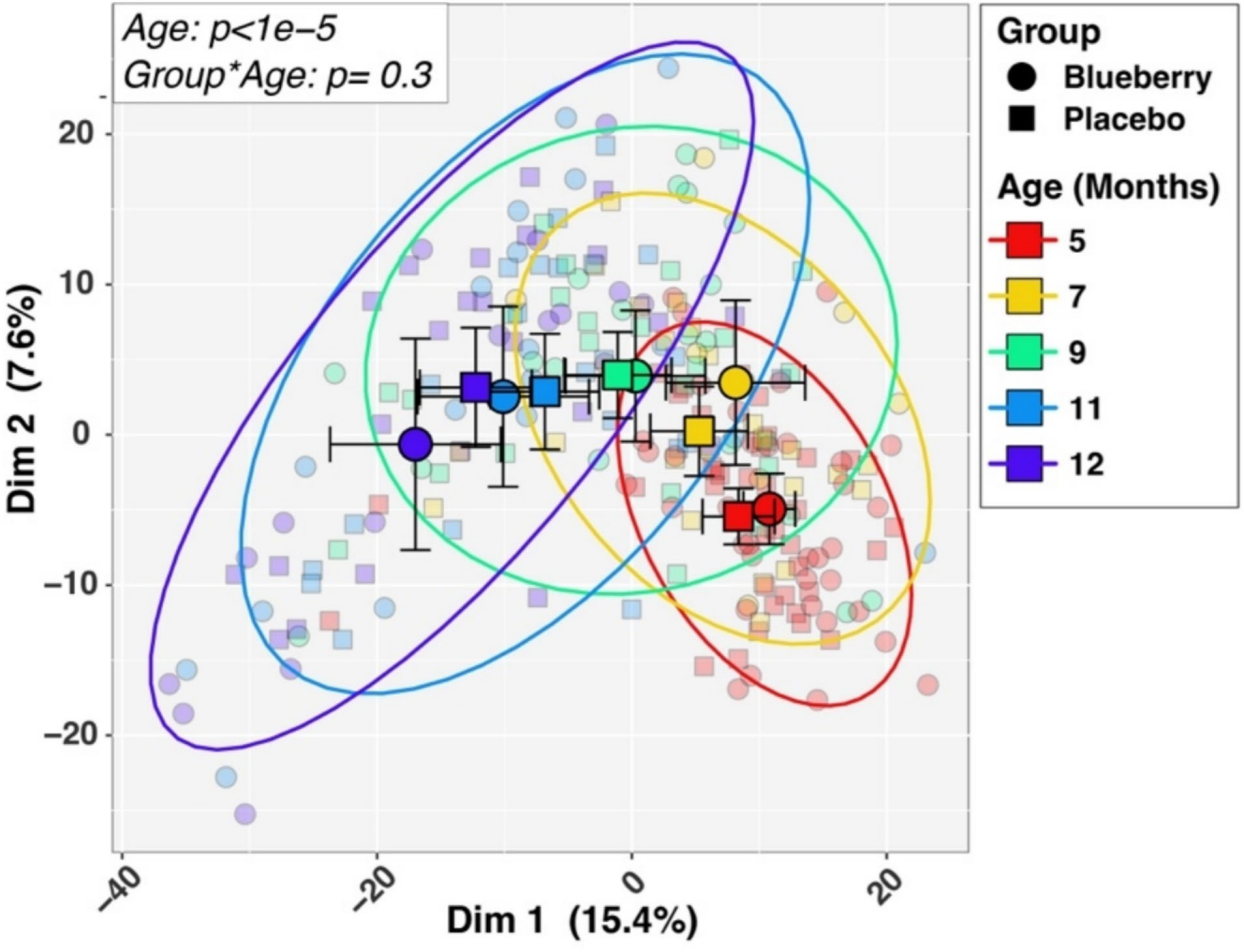
Longitudinal changes in beta-diversity. Differences in microbiotas by age and dietary group in beta-diversity, differences in microbiota composition among different groups, are visualized by Principal Coordinates Analysis plot of the first two principal components. Larger symbols represent group means while smaller symbols represent individual samples. Group and age affiliations are indicated in the legend. *p-*values for Age and the Group by Age interaction term, shown in the inset box, were inferred by PERMANOVA analysis (see Methods).

Initial univariable and multivariable analyses of beta-diversity indicated that sequencing batch (*p* = 0.0097) and mode of delivery (*p* = 0.034) were significantly associated with baseline microbiota composition, while other variables (sex, ethnicity, antibiotics, mode of feeding, formula consumption, percent compliance) were not significant. Therefore, all subsequent analyses (alpha-diversity, beta-diversity, differential abundance) included batch and mode of delivery as covariates in statistical tests. Measures of both alpha-diversity (richness [Sobs], evenness [Shannon H/Hmax], Shannon diversity (H); Figure 3) and beta-diversity (Figure 4) changed significantly with age. For instance, all three alpha-diversity indices increased with age (*p* < 0.001; Figure 3). Group-by-age interaction terms for richness and Shannon diversity had *p*-values of 0.087 and 0.085, respectively, indicating a trend for a modifying effect of blueberry powder on age-dependent changes in alpha diversity. In both cases, the blueberry powder diet increased alpha-diversity compared with placebo diet (Figure 3). In contrast, for beta-diversity neither Group-by-Age interaction terms nor Group main effects were significant in either longitudinal (Figure 4) or cross-sectional analyses.

### Gut microbial taxa

**Figure 5:**
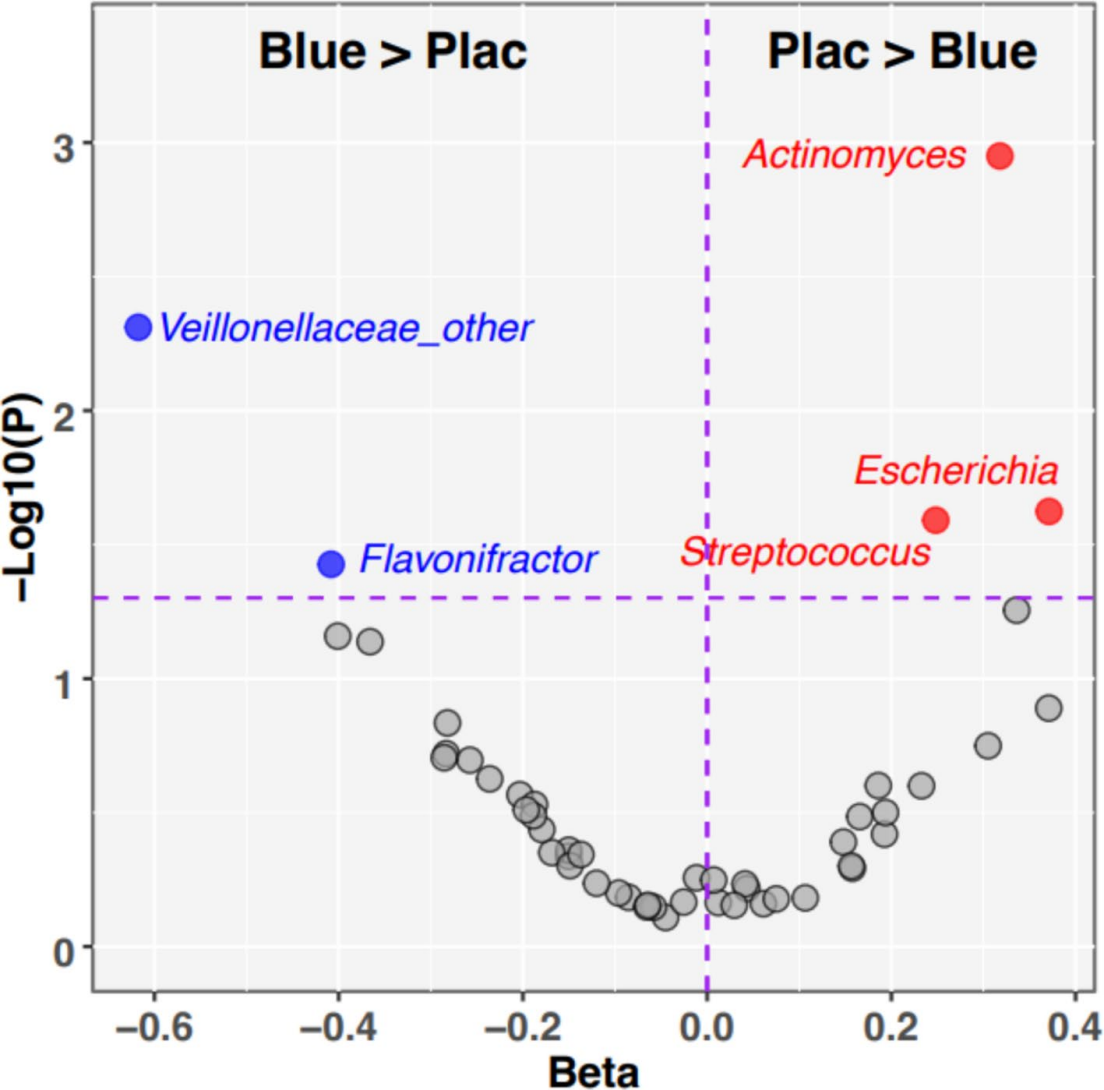
Individual taxa with significant Dietary Group by Age interactions. The volcano plot summarizes the results of linear mixed effects modeling of taxon relative abundances that assessed Diet*Age interaction terms. Taxa in the upper left quadrant and colored blue were significantly enriched (*p* < 0.05) in blueberry group compared to placebo. Taxa in the upper right quadrant and colored red were significantly enriched (*p* < 0.05) in placebo relative to blueberry diet. Taxa that did not meet the significance cutoff are colored gray. *X*-axis: −1*Log10 (*p*-value). *Y*-axis: regression Beta for Group*Age interaction term.

Next, we identified individual taxa whose relative abundances exhibited Group-by-Age interactions (Figure 5). The blueberry group increased the abundances of four taxa over time, compared with the placebo: *Veillonaceae, Flavonifractor, Subdoligranulum,* and *Butryicicoccus*. Conversely, four taxa decreased in abundance on the blueberry powder diet relative to the placebo: *Actinomyces, Escherichia, Streptococcus,* and *Romboutsia.* Of these, *Actinomyces* had an FDR-corrected *p*-value which was trended towards significance (*p*-adjusted = 0.056). The relative abundances of taxa between groups at 12 months at phylum, family and genus levels (Supplementary Figure 1).

## Discussion

To our knowledge, this is the first double-blind, randomized, placebo-controlled trial to examine changes in the gut microbiota associated with blueberry powder consumption during complementary feeding of U.S. infants. Overall, findings demonstrated that introducing blueberry powder as a first complementary food is feasible and may exert additive benefits on top of the well-documented benefits of breastfeeding on gut microbiota development (42, 43), supporting our overall hypothesis. Growth was not expected to differ between groups as the base diet of the participants was not controlled, and the blueberry powder only added a very small amount of energy to the complementary diet.

In this cohort, both the blueberry and placebo groups had an increase in gut microbial alpha diversity. As reported previously, infant age, or the introduction of complementary foods, usually led to an increase in alpha diversity (7, 44), which may be interpreted as increased microbial stability and progression toward a more mature gut microbiota (45). In addition, the blueberry group tended to have a greater increase in alpha-diversity compared to the placebo. This trend is consistent with previous works in adults and animal models that adding blueberries to the diet increases gut microbiota alpha diversity (46). The lack of overall significance in this cohort could be due to the relatively small sample size. In adults, alpha-diversity is an indicator for metabolic and gastrointestinal health and low alpha-diversity have been reported to be associated with obesity and metabolic syndromes (47, 48). In terms of gut microbial taxa, two taxa - *Veillonaceae and Flavonifractor -* were more abundant in the blueberry group. Some studies showed that the *Veillonaceae* family helps metabolize lactate, preventing lactate accumulation and thus contributing to a balanced gut microbiome (49), although results are inconsistent (50–52). It may also serve a protective role in the development of childhood asthma development (53). *Flavonifractor* exhibits anti-inflammatory and antioxidant properties through flavonoid degradation (54). Metabolites from both genera have been detected in stool samples of human milk-fed infants (55). *Subdoligranulum* may also suppress development of food allergy in infants, though the research is limited (56).

Besides these taxa, the blueberry group also had a trended decrease in *Actinomyces, Escherichia, and Streptococcus. Escherichia* and *Streptococcus* can be considered potential pathogens (57). In infants with eczema, higher levels of *Escherichia* have been reported (58), with the genus correlating with total serum IgE (59). *Actinomyces,* generally regarded as a commensal bacterium, has also been found in greater abundance in infants later diagnosed with allergic diseases (60). As the current findings were marginally non-significant, they should be considered preliminary, and more in-depth investigations need to be done before definitive recommendations can be made.

The liquid diet (human milk and formula) was not considered in the dietary analysis for two main reasons. First, the aim of this study was to investigate the potential benefits of adding blueberry powder as one of the first complementary foods. Thus, the focus was on complementary diets, which are more subjective to caregiver’s choice and have more potential for improvement. Second, human milk intake could not be quantified with nursing duration, as recorded in the diet records. Given that the groups had similar formula intake, we infer that human milk intake did not differ between groups and therefore did not confound the statistical analysis and internal validity of the findings, however this is an important limitation to consider. The diet record analysis did show that energy and macronutrient intakes from complementary foods, and growth trajectories, were comparable between groups. Although growth z-scores did not differ between groups or longitudinally, participants demonstrated an overall lower than population median WAZ and LAZ, which persisted throughout the study, which is consistent with other studies describing Colorado infants (61, 62).

Although this study was rigorously designed, it has several limitations. First, participants were provided with blueberry/placebo packets and requested to avoid other blueberries and blackberries for the duration of the study; however, their diet was otherwise not controlled. Due to these analyses being completed in the context of a complex diet, other dietary factors may also be at play, although the initial diet records did not show group differences. One such factor is infant formula. While we did not observe differences in formula consumption at baseline or 12 months between groups, we did not collect information on brand, timing, or amounts of formula prior to enrollment, which may potentially impact the baseline microbiome and growth of infants. Second, the study was from 5 to 12 months of age, and follow-ups of these infants past one year to determine the long-term effects of early blueberry powder consumption on infant health outcomes were not completed. If possible, future work with follow-up beyond 1 year is warranted. Third, our analyses do not allow generalization to less frequent blueberry consumption, nor any potential dose-dependent effects. As such, it would be beneficial to determine the amount and frequency of blueberry consumption at which the observed changes are detected, including offering fresh or frozen blueberries compared to a freeze-dried powder. Lastly, the sample size is relatively small, which could have contributed to certain trends towards significance observed.

Full compliance was defined as consuming one packet of the provided powder, blueberry or placebo, per day. Compliance in the blueberry group was lower compared to the placebo, partially due to the challenges caregivers encountered when mixing the freeze-dried blueberry powder with other baby foods or human milk. Each packet of the freeze-dried blueberry powder, or 2 ounces of fresh blueberries, is equivalent to 5 servings of fruit for infants (17, 18). Thus, although compliance was low (37.4% at 11-months and 36.8% at 12-months) for infants in the blueberry group, they still consumed, on average, at least one serving of blueberries per day, which increases the generalizability of the findings in the present study.

Overall, findings from this study suggest that blueberry powder serve as purees could be considered as one of the first complementary (solid) foods in weaning human-milk-fed infants, if properly introduced, with additive benefits in gut microbiota development and further support the effects of complementary feeding on the developing microbiome, assuming the liquid diet (human milk and infant formula) did not interact with blueberry on gut microbiota changes. Although current dietary guidance for infants acknowledges the importance of iron-and zinc-rich foods (e.g., meat) (63, 64), when it comes to fruits and vegetables, it does not differentiate among the sources of other nutrients and bioactive compounds (such as phytochemicals), in part due to the lack of evidence. This study provided valuable information that could potentially inform future recommendations for infants and toddlers on the selection of fruits and vegetables, and may include consideration of effects observed with the consumption of pureed blueberry powder, as well as complementary food choices beyond meeting the nutrient needs of growing infants (e.g., gut microbiota outcomes).

## Data Availability

The datasets presented in this study can be found in online repositories. The names of the repository/repositories and accession number(s) can be found at: https://www.ncbi.nlm.nih.gov/, PRJNA1245476.

## Supplementary material

SUPPLEMENTARY FIGURE 1 Relative abundances of bacterial taxa grouped by diet. Stacked bar charts display mean percent relative abundances (%RA) of taxa between diets, organized at phylum, family, and genus levels. Taxa with mean %RA less than 0.5% were aggregated into the “Other” category.
