# Supplementary figures and images for: Introducing blueberry powder as one of the first complementary foods changes the gut microbiota composition and diversity in U.S. human milk-fed infants: a double-blind, randomized controlled trial
Source: Front Nutr. 2025 Sep 4;12:1623521. doi: 10.3389/fnut.2025.1623521 (PMC12445049; doi:10.3389/fnut.2025.1623521)

## Phylum

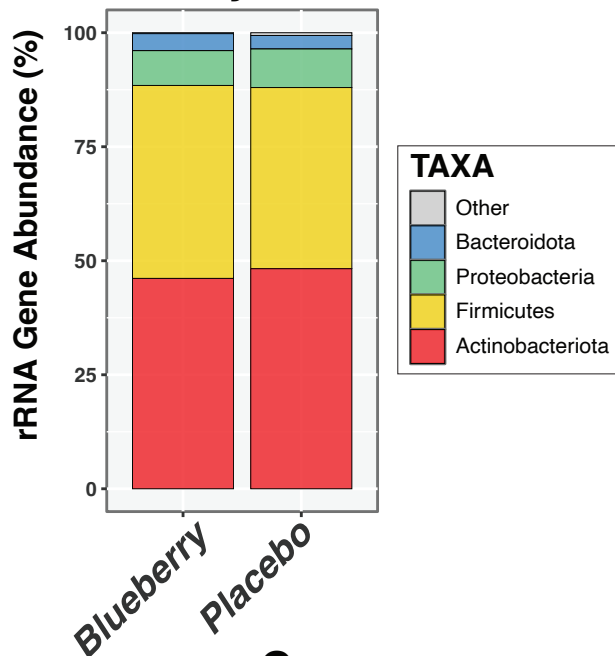

## Family

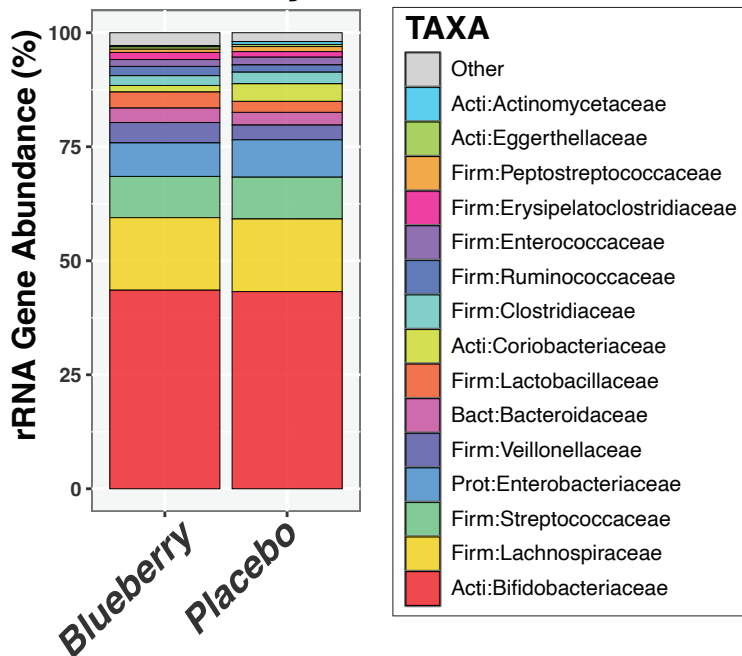

## Genus

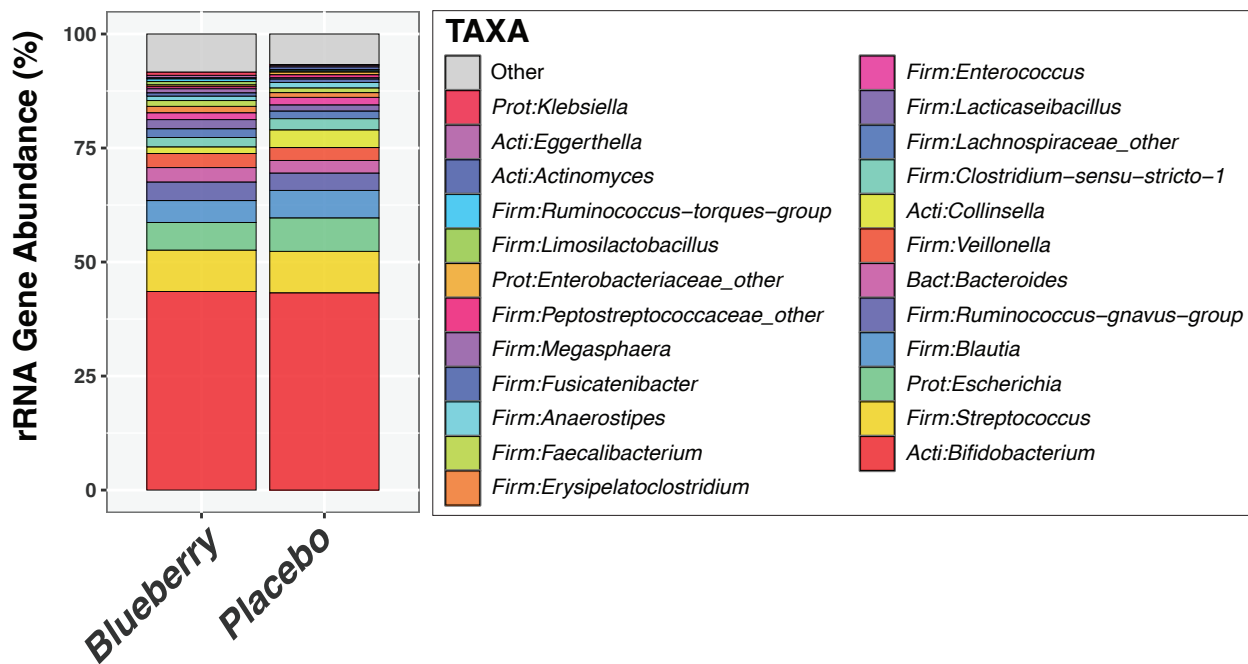

Supplement: SUPPLEMENTARY FIGURE 1 — Relative abundances of bacterial taxa grouped by diet. Stacked bar charts display mean percent relative abundances (%RA) of taxa between diets, organized at phylum, family, and genus levels. Taxa with mean %RA less than 0.5% were aggregated into the “Other” category. [file Image_1.pdf]
