# Supplementary material for: Introducing blueberry powder as one of the first complementary foods changes the gut microbiota composition and diversity in U.S. human milk-fed infants: a double-blind, randomized controlled trial
Source: Front Nutr. 2025 Sep 4;12:1623521. doi: 10.3389/fnut.2025.1623521 (PMC12445049; doi:10.3389/fnut.2025.1623521)
Supplement: Supplementary file 2 [file Table_1.docx]

Supplementary Table: Nutrient breakdown of freeze-dried blueberry powder and placebo powder (per 10 g packet)

| **Nutrients** | **Blueberry Powder** | **Placebo Powder** |  |
| --- | --- | --- | --- |
|  |  |  |  |
| **Calories** | 39.6kcal | 36.2kcal |  |
| **Fat** | 0.120g | 0.267g |  |
| **Sodium** | <0.30mg | 2.12mg |  |
| **Carbohydrates** | 9.3g | 9.0g |  |
| **Fiber** | 2.22g | 0.0964g |  |
| **Dextrose** | 2.78 | 3.54 |  |
| **Glucose** | 3.06 | 3.25 |  |
| **Protein** | 0.237g | 0.041g |  |
| **Vitamin C** | 0.576mg | <0.10mg |  |
| **Calcium** | 4.03mg | 1.43mg |  |
| **Potassium** | 47.8mg | 3.70mg |  |
