# Supplementary material for: Introducing blueberry powder as one of the first complementary foods changes the gut microbiota composition and diversity in U.S. human milk-fed infants: a double-blind, randomized controlled trial
Source: Front Nutr. 2025 Sep 4;12:1623521. doi: 10.3389/fnut.2025.1623521 (PMC12445049; doi:10.3389/fnut.2025.1623521)
Supplement: Supplementary file 3 [file Table_2.docx]

| 12 months | Blueberry (n=30) | Placebo (n=31) | p-value |
| --- | --- | --- | --- |
| Received formula, n(%) | 12 (40%) | 7 (23%) | 0.14 |
| Ounces of formula, mean ± SD | 13.8 ± 5.3 | 16.5 ± 9.7 | 0.44 |

Supplemental Table 2: Formula consumption between groups.

Table reflects formula intake between groups at 12 months. SD: standard deviation. The number of participants who received formula in the preceding month (12-months) is presented as n(%). The average ounces of formula consumed by participants is presented as mean ± SD. Significance was set at p<0.05.
